# Trabecular bone score in the assessment of bone health in thalassemia major

**DOI:** 10.3389/fendo.2025.1635068

**Published:** 2025-11-18

**Authors:** Martina Di Noto, Anastasia Xourafa, Rosamaria Rosso, Anna Bulla, Antonino Catalano, Federica Bellone, Luca Zanoli, Enrico Buccheri, Pietro Castellino, Agostino Gaudio

**Affiliations:** 1 Department of Clinical and Experimental Medicine, University of Catania, Catania, Italy; 2 Unità Operativa Semplice Dipartimentale (UOSD) Talassemia, Azienda Ospedaliera Universitaria (AOU) Policlinico “G. Rodolico – San Marco”, Catania, Italy; 3 Department of Clinical and Experimental Medicine, University of Messina, Messina, Italy; 4 Unità Operativa Complessa (UOC) Medicina Interna, Azienda Ospedaliera Universitaria (AOU) Policlinico “G. Rodolico – San Marco”, Catania, Italy

**Keywords:** TBS, thalassemia major, osteoporosis, bone health, DXA

## Abstract

**Introduction:**

Thalassemia major (TM) has a significant impact on patients’ quality of life. Osteoporosis and osteopenia are common and important complications, yet they are often underestimated. Their etiology is multifactorial, culminating in reduced bone mass, impaired remodeling, and increased fracture risk. The reported prevalence of osteopenia and osteoporosis in TM is highly variable, reaching up to 90%. The trabecular bone score (TBS) is a relatively recent analytical tool that measures lumbar spine texture on dual X-ray absorptiometry (DXA) images. At present, only limited data are available on its use in TM. The aim of this retrospective study was to assess bone health in TM and to evaluate the TBS as a potential diagnostic and prognostic tool.

**Methods:**

Eighty-eight adult outpatients with TM (mean age: 41.9 ± 8.9 years) were enrolled. The following parameters were evaluated: laboratory tests, DXA measurements, and thoracic–lumbar spine X-rays (assessed by Genant’s method).

**Results:**

Reduced BMD was observed in 96.3% of patients. TBS values were also markedly reduced; however, their performance in discriminating fractures in this cohort appeared to have limited clinical utility.

**Discussion:**

The TBS may be a valuable, non-invasive complementary tool for assessing bone quality alongside BMD, although it does not reliably discriminate between patients with and without fractures. Further investigations are needed to clarify the role of the TBS in predicting fracture risk in TM.

## 1 Introduction

Beta-thalassemias are a group of hereditary disorders characterized by altered synthesis of hemoglobin (Hb) beta chains. Based on the increasing severity of the resulting clinical presentation, three main forms have been described: thalassemia major (TM), thalassemia intermedia (TI) and thalassemia minor. TM has a significant impact on the patient’s life because transfusion-dependent anemia is burdened with several complications due to iron overload, specifically growth retardation and endocrine gland involvement (hypogonadism, diabetes mellitus, and insufficiency of the thyroid, parathyroid and pituitary glands), chronic hepatitis and hypersplenism, dyspnea, and dilated cardiomyopathy (1).

Although optimization of transfusion regimens and iron-chelation therapy has greatly increased the life expectancy of patients with thalassemia and improved their quality of life, osteoporosis and osteopenia remain serious and common complications (2). Bone alterations arise from a complex interplay of factors, including anemia, iron overload, deferoxamine toxicity, and insulin growth factor-1 deficiency, and are worsened by hypogonadism and bone marrow expansion (3–5). In addition, alterations in the receptor activator of NF-κB (RANK)/receptor activator of NF-κB ligand (RANKL)/osteoprotegerin system and other cytokines may act as mediators of increased bone resorption (6, 7). There is also evidence suggesting that genetic factors, such as vitamin D receptor (*VDR*) and collagen type 1 alpha 1 (*COL1A1*) gene polymorphisms, are related to the development of this complication (8).

In patients with TM, low bone mineral density (BMD) values are very common (9, 10). In the literature, the reported prevalence of osteopenia and osteoporosis in TM is highly variable—up to 90%—even among optimally transfused patients (with pre-transfusion Hb levels of 9.0–9.5 g/dL) who are well chelated as adults. This wide variability depends largely on the densitometric methods used and the criteria for patient selection (3).

Bone impairment in patients with TM is associated with an increased risk of fractures. Recent studies have shown fracture rates ranging from 16.6% to 49.1%, depending on the population studied and the types of data analyzed (3). Some authors noted that the bimodal fracture distribution described in the general population—sports-related in adolescents and osteoporosis-related in postmenopausal women—was not observed in patients with thalassemia. This absence may be explained by the cross-sectional design of the studies or by decreased physical activity in this group (9). Individuals with thalassemia who experience fractures tend to have lower BMD T-scores and Z-scores (9, 11) and are more likely to be hypogonadal and dependent on hormone replacement therapy (12).

The main goals in managing bone damage are pain control, improvement of BMD, and reduction of fracture risk. Strategies proposed include the use of bisphosphonates (13–18), strontium ranelate (19), or denosumab (20), along with vitamin D and calcium supplementation, diabetes management, hormone replacement therapy, iron chelation, and normalization of Hb levels (5, 13, 21).

Given the high prevalence of bone involvement and its significant impact on quality of life, a detailed assessment of bone health in patients with thalassemia is essential. To date, however, estimating the true prevalence of bone impairment in this population remains a major challenge.

The trabecular bone score (TBS) is a relatively recent analytical tool that performs texture measurements on lumbar spine dual X-ray absorptiometry (DXA) images, capturing information on trabecular microarchitecture. The TBS is calculated using commercially available add-on software from standard DXA images. A low TBS indicates degraded bone microarchitecture and may help predict fracture risk, partially independent of BMD. The TBS has been successfully applied in various forms of osteoporosis (e.g. in post-menopausal women, patients with diabetes, individuals receiving glucocorticoid therapy, patients with endogenous Cushing’s syndrome, those on hemodialysis, and breast cancer survivors taking aromatase inhibitors), particularly as a complement to BMD. Other studies have evaluated the TBS in conditions such as primary hyperparathyroidism, acromegaly, anorexia nervosa, primary aldosteronism, rheumatic diseases, differentiated thyroid carcinomas, Ehlers–Danlos syndrome, and thalassemia (22–25). However, data regarding thalassemia remain limited, and the role of the TBS in this population is still under investigation.

The aim of this retrospective study was to assess the impact of TM on bone health and to evaluate the TBS as a potential diagnostic and prognostic tool in this group of patients.

## 2 Materials and methods

This retrospective study involved 88 adult outpatients (43 women and 45 men) aged 25–60 years (mean age: 41.9 ± 8.9 years). All patients were affected by TM and were followed at the U.O.S.D. Thalassemia of the ‘G. Rodolico’ Hospital in Catania. Data were extracted from clinical records. All patients received standard transfusion support and iron chelation therapy according to Institute protocols, along with treatments for specific complications (e.g. hormone replacement therapy, vitamin D supplements with cholecalciferol to maintain serum 25-OH vitamin D levels above 30 ng/mL, and/or bisphosphonates).

The exclusion criterion was previous use of active bone agents (except bisphosphonates), including denosumab, selective estrogen receptor modulators, and strontium ranelate.

The following parameters were evaluated: anthropometric characteristics, medical history (including prior fragility fractures), systemic complications, iron-chelating therapy, and mean Hb and ferritin values. Height and weight were measured at baseline according to standard procedures, and body mass index (BMI) was calculated as weight in kilograms divided by the square of height in meters (kg/m^2^). Fragility fractures were defined as any fractures resulting from low-level (or low-energy) trauma, such as falling from standing height or less (26). Fracture prevalence was determined using both medical records and radiographic assessment of the spine. Endocrinopathies were defined as the presence of at least one of the following endocrine disorders: hypogonadism, diabetes mellitus, hypothyroidism, or hypoparathyroidism.

Where available, a lateral thoracic and lumbar spine X-ray image was used to evaluate morphometric vertebral fractures, which were diagnosed when a vertebral body showed at least a 20% reduction in anterior, middle, or posterior height compared with the same or adjacent vertebra (27).

As reported in one of our previous studies (28), BMD was measured with a DXA densitometer (enCORE version 16, GE Lunar Prodigy; GE Medical Systems, Chicago, IL, USA), both at the lumbar spine (L1–L4) in anterior–posterior projection and at the femoral neck. The instrument was calibrated daily according to the manufacturer’s instructions. Reproducibility was calculated as a coefficient of variation (CV), obtained from weekly measurements of a standard phantom on the instrument and from repeated measurements in three patients of different ages. The CV of our instrument was 0.5% with the standard phantom; *in vivo*, we calculated a CV of 1.1% for the lumbar spine and 1.5% for the femoral neck. BMD data are expressed in g/cm^2^, T-score and Z-score. Diagnosis of osteoporosis and osteopenia was made according to World Health Organization (WHO) criteria (29).

In addition, we investigated the TBS through further evaluation of the available DXA images using TBS iNsight software version 3.0.3.0 (Medimaps Group, Canejan, France). The TBS was calculated from the variogram of the trabecular bone–projected image, defined as the sum of the squared grey-level differences between pixels at a specific distance and angle. The TBS was then obtained as the slope of the log–log transform of this variogram (30). We used Cormier’s proposed TBS cut-offs (TBS of ≥1.350 indicating normal bone tissue, TBS of ≤1.200 indicating degraded bone tissue, and TBS of 1.200–1.350 indicating partially degraded bone in postmenopausal women) (31).

Data for continuous variables are expressed as mean ± standard deviation (SD) for normally distributed data, while median and interquartile range were used for non-normally distributed data. Categorical variables are presented as percentages. Normal distribution of values was verified for the different parameters using the Shapiro–Wilk test. Pearson linear regression analysis (normal distribution) or the Spearman test (non-normal distribution) was applied for association studies. Comparisons of continuous variables between groups were made using Student’s t test (normal distribution) or the Mann–Whitney test (non-normal distribution). Comparisons of categorical variables between groups were performed using the χ^2^ test. Receiver operating characteristic (ROC) curves were constructed, and the area under the curve (AUC) was calculated to evaluate the performance of different parameters in discriminating between patients with and without fractures. A p value of 0.05 was considered statistically significant. Descriptive statistics and significance levels were analyzed using JASP Statistics for Windows, Version 0.19.1.0.

The protocol was approved by the local ethics committee (Comitato Etico Catania 1, Azienda Ospedaliero–Universitaria Policlinico ‘G. Rodolico – San Marco’ Catania), approval number 24, on 8 January 2024.

## 3 Results

**Table 1** shows the clinical, densitometric, and laboratory parameters of patients with TM. There were no statistically significant differences between men and women in age, mean Hb values, BMD at the lumbar spine (L1–L4) and femoral neck, or TBS. However, the two groups differed significantly in BMI (22.29 ± 2.99 kg/m^2^ for women vs 24.16 ± 3.76 kg/m^2^ for men, p = 0.011). All patients had BMI values within the accuracy range for TBS analysis reported by Medimaps (15–35 kg/m^2^).

**Table 1:** Clinical, densitometric, and laboratory data in men and women with TM.

	Total	Men	Women	P-value
n	88	45	43	
Age (years)	43.5 (13.5) ^d^	43.2 (9.1) ^c^	40.5 (8.6) ^c^	0.155
BMI (Kg/m^2^)	22.76 (4.50) ^d^	24.16 (3.76) ^c^	22.29 (2.99) ^c^	0.011
Lumbar BMD (g/cm^2^)	0.853 (0.156) ^d^	0.869 (0.140) ^d^	0.837 (0.178) ^d^	0.277
Lumbar T-score	-2.8 (1.38) ^d^	-2.6 (1.4) ^d^	-3.0 (1.2) ^d^	0.472
Lumbar Z-score	-2.7 (1.20) ^d^	-2.7 (1.0) ^d^	-2.6 (1.2) ^d^	0.398
Femoral BMD (g/cm^2^)	0.760 (0.160) ^d^	0.788 (0.158) ^d^	0.715 (0.142) ^d^	0.064
Femoral T-score	-2.1 (1.2) ^d^	-2.0 (1.3) ^d^	-2.3 (1.0) ^d^	0.096
Femoral Z-score	-1.8 (1.2) ^d^	-1.8 (1.2) ^d^	-1.9 (1.2) ^d^	0.831
TBS	1.134 (0.142) ^d^	1.128 (0.176) ^d^	1.160 (0.120) ^d^	0.763
Hemoglobin (g/dL)	9.70 (0.71) ^d^	9.80 (0.68) ^d^	9.67 (0.72) ^d^	0.643
Ferritin (ng/mL)	398.45 (729.32) ^d^	384.22 (626.51) ^d^	450.33 (757.59) ^d^	0.415
Fractures n (%) *^a^*	44 (50%)	25 (55.5%)	19 (44.1%)	0.286
Endocrinopathies n (%) *^b^*	31 (35%)	18 (40%)	13 (30%)	0.337

^a^Vertebral fractures only (n = 32); vertebral plus other sites (n = 4); upper extremity fractures (n = 3); lower extremity fractures (n = 3); combined upper and lower extremity fractures (n = 2).

^b^Hypogonadism (n = 20), hypothyroidism (n = 11), diabetes mellitus (n = 6), and hypoparathyroidism (n = 1); 6 patients presented multiple endocrine abnormalities.

Continuous variables are expressed as ^c^mean (SD) or ^d^median (IQR).

According to the WHO classification, 96.3% of patients had reduced BMD (40.2% with osteopenia and 56.1% with osteoporosis). Women showed a higher prevalence of osteoporosis than men (62.5% vs 50.0%, p > 0.05), whereas osteopenia was more prevalent in men (47.6% vs 32.5%, p > 0.05). These differences were not statistically significant. Fragility fractures were reported in 44 patients (50%), including both vertebral and non-vertebral fractures (vertebral fractures alone, n = 32; vertebral plus other sites, n = 4; upper extremity, n = 3; lower extremity, n = 3; combined upper and lower extremities, n = 2). Although fractures were more frequent in men (55.5%) than in women (44.1%), this difference did not reach statistical significance. Endocrine disorders were identified in 31 patients: hypogonadism (n = 20), hypothyroidism (n = 11), diabetes mellitus (n = 6), and hypoparathyroidism (n = 1), with 6 patients presenting multiple endocrine abnormalities. The prevalence of endocrinopathies did not differ between men and women.

**Table 2** shows the clinical, densitometric, and laboratory parameters in patients with and without fractures.

**Table 2:** Clinical, densitometric, and laboratory data in patients with and without fractures.

	Fractures	Non-fractures	P-value
n	44	44	
Age (years)	43.0 (8.7) ^a^	40.8 (9.1) ^a^	0.229
Sex (M/F)	(25/19)	(20/24)	0.286
BMI (Kg/m^2^)	22.76 (3.69) ^b^	22.81 (4.93) ^b^	0.983
Lumbar BMD (g/cm^2^)	0.803 (0.107) ^b^	0.924 (0.154) ^b^	<0.001
Lumbar T-score	-3.1 (0.8) ^a^	-2.2 (1.3) ^a^	<0.001
Lumbar Z-score	-3.1 (0.8) ^a^	-2.0 (1.3) ^a^	<0.001
Femoral BMD (g/cm^2^)	0.705 (0.119) ^b^	0.818 (0.145) ^b^	0.0014
Femoral T-score	-2.3 (0.8) ^b^	-1.8 (1.3) ^b^	0.003
Femoral Z-score	-2 (0.8) ^b^	-1.3 (1.3) ^b^	<0.001
TBS	1.100 (0.161) ^b^	1.171 (0.149) ^b^	0.052
Hemoglobin (g/dL)	9.81 (0.67) ^b^	9.60 (0.77) ^b^	0.074
Ferritin (ng/mL)	314.76 (404.77) ^b^	519.50 (810.79) ^b^	0.014
Endocrinopathies n (%)	19 (44.1%)	12 (27.2%)	0.118

Continuous variables are expressed as ^a^mean (SD) or ^b^median (IQR).

Patients with fractures had significantly lower lumbar and femoral BMD values than those without fractures (lumbar BMD: 0.803 ± 0.107 vs 0.924 ± 0.154 g/cm^2^, p < 0.0001; femoral BMD: 0.729 ± 0.126 vs 0.815 ± 0.128 g/cm^2^, p = 0.003). There were no statistically significant differences between the two groups in age, sex, BMI, Hb levels, or the presence of endocrinopathies. Ferritin levels were lower in patients with than without fractures (314.76 ± 404.77 vs 519.50 ± 810.79 ng/mL, p = 0.014).

**Figure 1:**
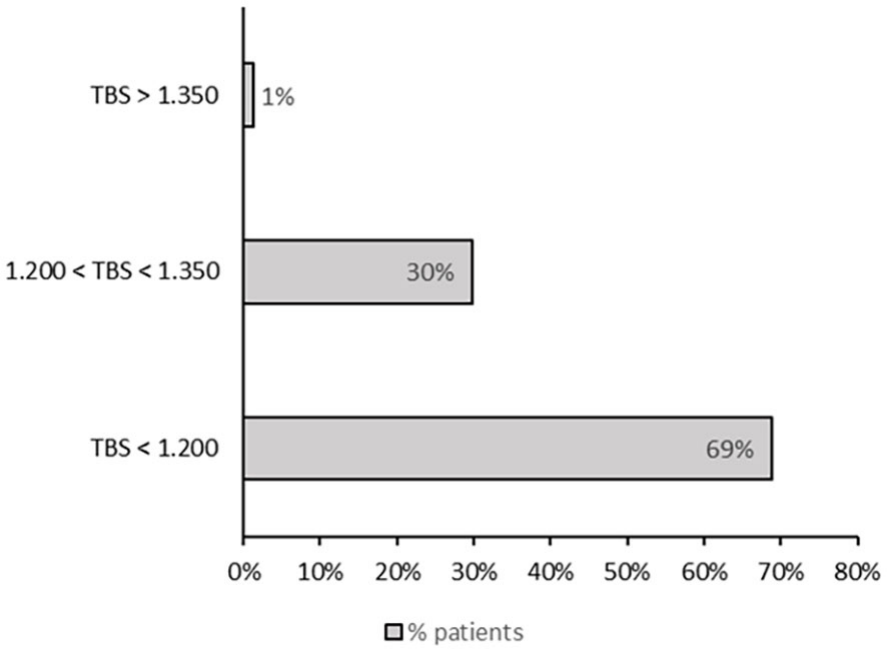
Distribution of patients according to TBS categories, based on the cut-off values proposed by Cormier et al. (31).

The TBS values in our cohort of patients with thalassemia were markedly reduced. **Figure 1** shows the distribution of patients stratified by TBS categories, based on the cut-off values proposed by Cormier et al. Fifty-three patients (69%) had a TBS of <1.200, 23 (30%) had a TBS of 1.200–1.350, and only 1 (1%) had a TBS of >1.350. TBS values were lower in patients with than without fractures, but this difference was at the threshold of statistical significance. Conversely, the difference between patients with osteopenia and osteoporosis was statistically significant (1.166 ± 0.107 vs 1.100 ± 0.138, respectively; p = 0.029).

**Figure 2:**
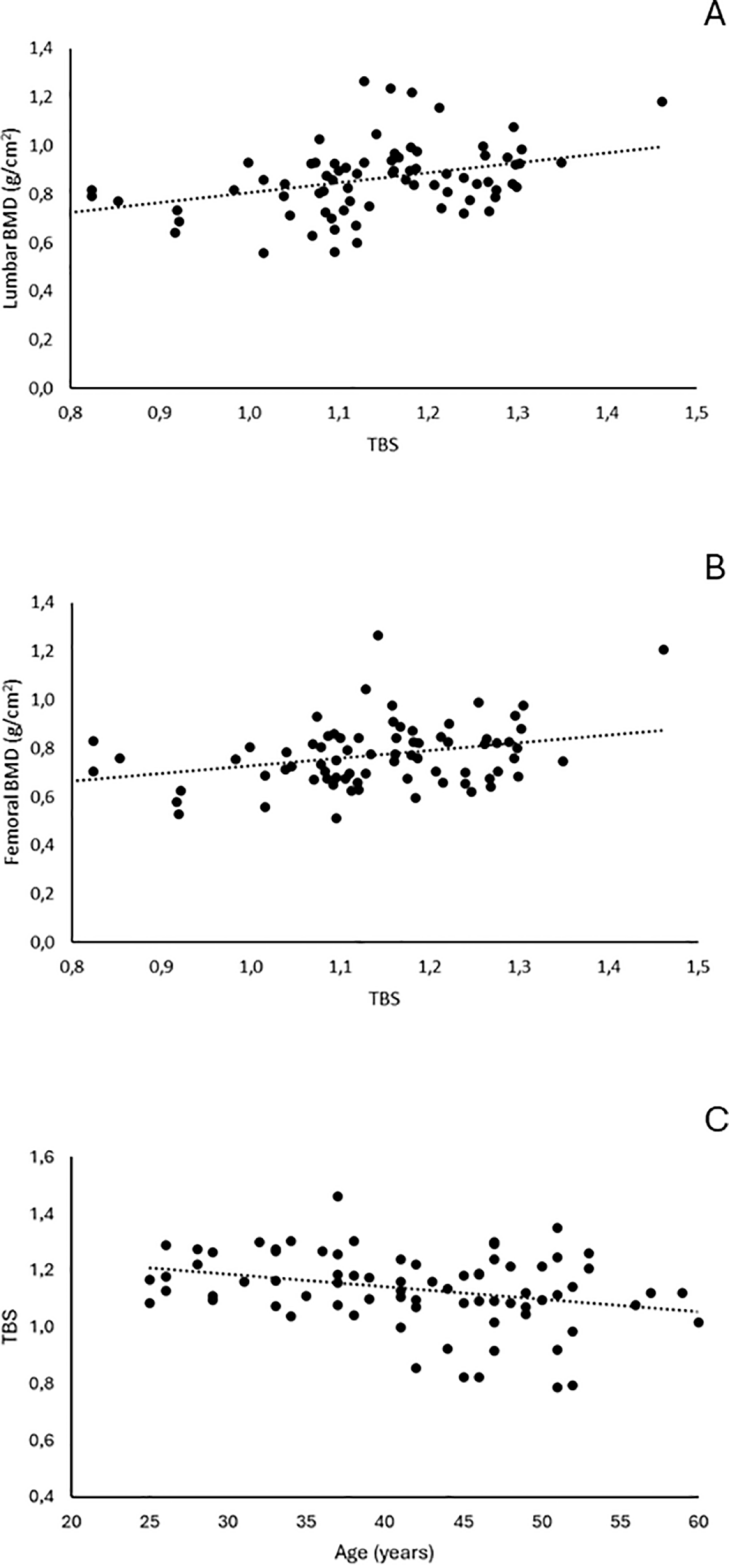
Correlation between TBS and **(A)** lumbar BMD values, **(B)** femoral BMD values, and **(C)** age.

TBS values correlated significantly with BMD values at the lumbar spine (r = 0.374, p = 0.0008) and femoral neck (r = 0.309, p = 0.006), as well as with age (r = −0.287, p = 0.011) (**Figure 2**).

**Figure 3:**
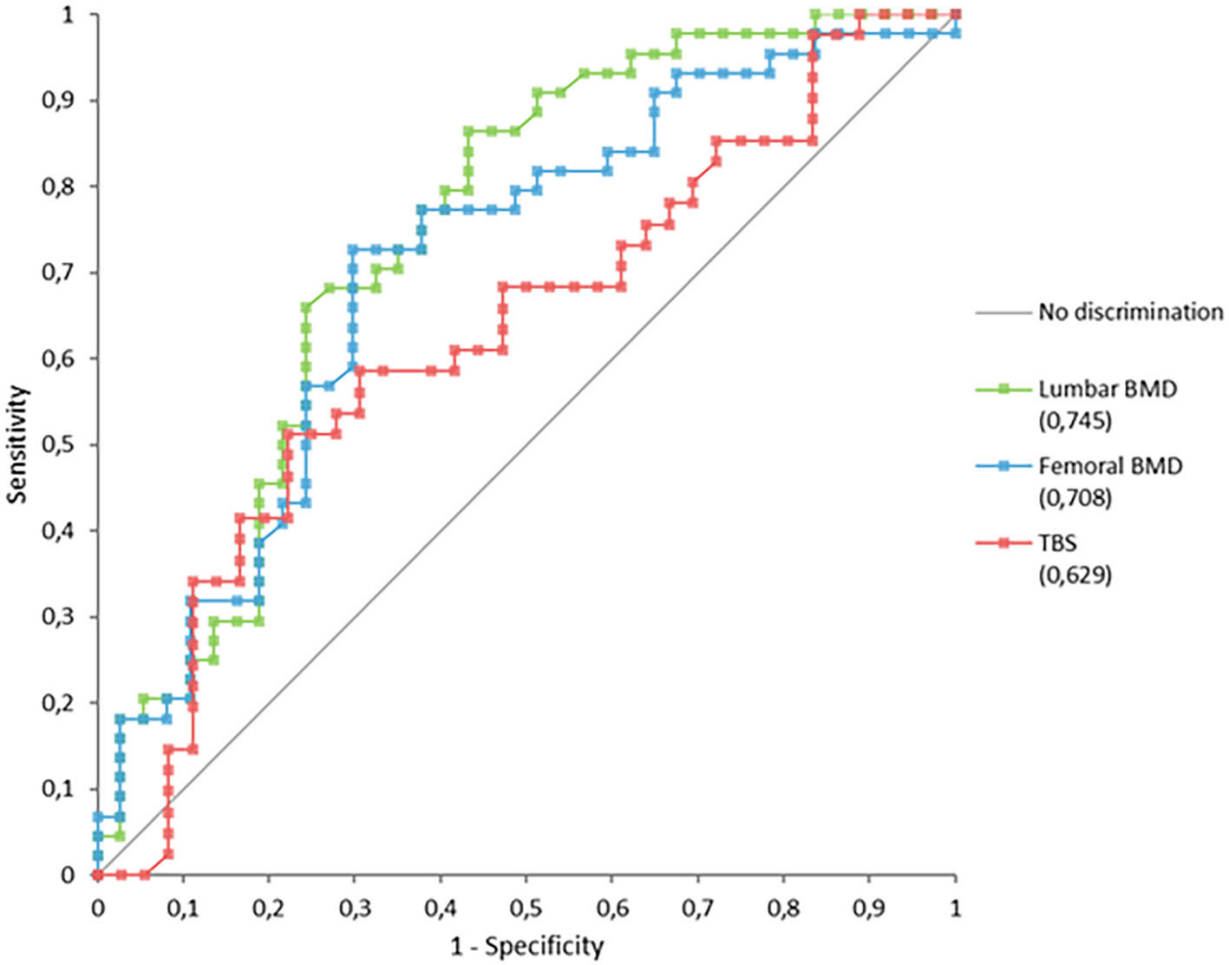
ROC curves for predicting fragility fractures in patients with thalassemia.

Analysis of ROC curves (**Figure 3**) showed that lumbar and femoral BMD values discriminated more effectively between patients with and without fractures than did the TBS (**Table 3**).

**Table 3:** AUCs in the prediction of fracture.

	AUC	95% CI	P-value
Lumbar BMD	0.745	0.633 to 0.856	<0.0001
Femoral BMD	0.708	0.592 to 0.823	0.0004
TBS	0.629	0.502 to 0.757	0.046

## 4 Discussion

Our data show that the prevalence of reduced BMD was 96.3% (40.2% in osteopenia and 56.1% in osteoporosis), according to the WHO classification. As highlighted in the literature, the reported prevalence of bone complications in patients with thalassemia is highly variable (3), depending on study population, diagnostic criteria, and age distribution. Our findings are consistent with published data, although they lie at the upper end of the reported spectrum. This high prevalence may be partly explained by the specific characteristics of our cohort, although we cannot exclude the influence of selection bias inherent to a retrospective, single-center study.

The prevalence of thalassemia-associated osteoporotic fractures has not been well established. In our study, the prevalence of fragility fractures was 50%, higher than that reported by Ruggiero (32) but comparable to the findings of Vogiatzi in patients with TM (9) and of Sutipornpalangkul et al. (33), who observed a prevalence of 44.1% in Thai patients with thalassemia. Discrepancies in prevalence across studies may be explained by two main factors: differences in the diagnostic methods used for vertebral fractures (such as self-reported fracture history questionnaires) and ethnic differences in fracture risk.

Endocrine disorders were identified in 31 patients. The most frequent was hypogonadism (n = 20), followed by hypothyroidism (n = 11), diabetes mellitus (n = 6), and hypoparathyroidism (n = 1). Among these, six patients presented multiple concomitant endocrinopathies, underscoring the complex and multifactorial nature of endocrine involvement in thalassemia. In men, hypogonadism is not always readily evident; therefore, these data may not be entirely accurate. Moreover, not all patients were on replacement therapy. Because of the limited sample size and the considerable heterogeneity of endocrinopathies in terms of type, treatment status, and potential underdiagnosis, subgroup analyses based on these variables were not performed.

The significant difference in ferritin levels between patients with and without fractures is noteworthy. Its explanation is not entirely clear because better control of iron overload appears to be associated with a worse impact on bone health, perhaps due to chelation therapy (3).

As supported by our findings, bone impairment remains one of the most important complications in TM, clearly requiring a detailed assessment of bone health. However, in the absence of standardized methods for these patients, such assessment continues to be a major challenge.

In clinical practice, DXA measurements are widely used to evaluate bone health in patients with TM. According to Vogiatzi (9), there is a strong correlation between DXA-derived Z-scores and fracture risk in TM: for every 1-SD decrease in the spine or femur BMD Z-score, the mean fracture rate increases by 37% or 47%, respectively (p < 0.001 for both). Nevertheless, other studies have highlighted limitations of DXA in patients with thalassemia due to the peculiar characteristics of bone architecture and deformities associated with the disease (34). Moreover, DXA does not provide information about bone microarchitecture, which appears to play a key role in bone strength, independently of bone mass (35).

Alternative strategies for evaluating BMD in TM include quantitative computed tomography (QCT) and quantitative ultrasonography (QUS). Studies suggest that QCT detects a lower prevalence of osteoporosis than does DXA in patients with beta-TM (35, 36), while data on QUS in thalassemia remain inconsistent (37, 38). Calculation tools for fracture risk have not yet been validated in thalassemia. Nonetheless, clinical risk factors such as age, BMI, smoking, and alcohol consumption should logically be considered, despite the limited supporting evidence (13). An increase in bone turnover markers (BTM) reflects the high rate of bone resorption in patients with TM (9, 39, 40), particularly those with hypogonadism (39). Despite their negative correlation with BMD, it has not been demonstrated whether BTM can independently predict fracture risk (9). Further data are needed to clarify their role in monitoring bone health in TM.

It is well established that fragility fractures result from a combination of quantitative alterations and microarchitectural damage (41), making the assessment of bone microarchitecture an essential component of evaluating bone health (42).

The TBS, approved by the U.S. Food and Drug Administration in 2012, is a texture index derived from standard lumbar spine DXA images that provides information on bone microarchitecture independently of BMD. The TBS is calculated from the experimental (empirical) variogram, which relates the variance in grey levels of the DXA image as a function of distance (43). The value of the TBS originates from the slope of the log–log transform of the two-dimensional variogram, which expresses the degree of grey-level amplitude variation within the DXA image. The TBS is obtained using commercially available software applied prospectively or retrospectively to standard DXA images from modern fan-beam densitometers (44). Values are unitless: higher TBS values correspond to more homogeneously textured bone with low-amplitude fluctuations in photon absorption, whereas less well-textured bone produces higher amplitude fluctuations and lower TBS values. To date, no consensus has been reached regarding cut-off levels for TBS. Cormier proposed that values of ≥1.350 indicate normal bone tissue, values of ≤1.200 indicate degraded bone tissue, and values of 1.200–1.350 represent partially degraded bone in postmenopausal women (25, 31). The reported *in vivo* short-term precision of the TBS ranges from 1.1% to 1.9%, which is comparable to the precision of lumbar spine BMD in the same studies (0.9%–1.4%) (45–47). In addition to bone texture, some technical factors can influence TBS values, including the DXA scan acquisition mode, soft tissue composition, presence of vertebral fractures, and differences in resolution between scanners and densitometer manufacturers (48, 49). In particular, abdominal soft tissue thickness may interfere with interpretation because image noise can affect TBS evaluation.

The ability of the TBS to assess bone structure independently of BMD has led many authors to explore its role in fracture risk prediction. The TBS has recently been shown to predict fracture risk in different forms of osteoporosis, either as an adjunct to BMD (e.g. in patients with diabetes, patients receiving glucocorticoid therapy, or patients with endogenous Cushing’s syndrome) or independently (e.g. in post-menopausal women, hemodialysis patients, and breast cancer survivors receiving aromatase inhibitors) (22–25). A study by Hans et al. supports the added value of combining the TBS with BMD to assess fracture risk (50), while data from Leslie demonstrate its ability to predict major osteoporotic fractures independently of the FRAX score and BMD (51, 52). A later study by McCloskey et al. showed that lumbar spine texture analysis through TBS is a risk factor for both osteoporotic fractures and mortality, confirming that its predictive capacity is independent of FRAX and BMD (53).

The TBS is emerging as a suitable tool for assessing bone health in thalassemia, complementing DXA measurements. Recent literature supports its use in both TM and TI (42, 54–56). Teawtrakul et al. reported that low BMD and TBS values were significantly associated with vertebral fractures in patients with thalassemia, particularly those with β-thalassemia/Hb E who had endocrinopathies (55). Osella et al. suggested that the TBS is superior to BMD and Z-scores in predicting vertebral deformities in beta-TM (56). In addition, studies by Baldini indicated a possible role for TBS in fracture risk stratification in both TM (42) and TI (54).

Encouraged by previous findings, we evaluated the use of the TBS in assessing bone health in our cohort of 88 adult patients with TM. All patients had BMI values within the accuracy range reported by Medimaps (15–35 kg/m^2^), ensuring reliability of the measurements and limiting artifacts.

In our study, TBS values correlated with lumbar and femoral BMD and with age, indicating that thalassemia negatively affects bone quality, particularly as patients get older. These results are consistent with previous reports (42).

We found that TBS values differed significantly between patients with osteopenia and osteoporosis (p = 0.029). In the literature, Boutroy et al. (57) showed that the TBS better predicts fracture risk in women with BMD in the normal or osteopenic range than in osteoporotic women.

In our cohort, TBS values were lower in patients with than without fractures, although this difference was only at the threshold of statistical significance. Analysis of AUC curves confirmed the predictive value of the TBS, as it allowed discrimination between patients with and without fractures. However, its performance was inferior to that of lumbar and femoral BMD, with an AUC below 0.650. The wide confidence interval, resulting from the limited sample size, represents an important limitation. This suggests that the model’s performance might improve in a larger cohort, reaching values of greater clinical utility.

These findings support the view that fracture pathogenesis in thalassemia is multifactorial, influenced not only by bone density or microarchitecture, but also by interacting factors such as endocrine dysfunction, iron overload, chelation history, and other clinical variables. Moreover, another possible influencing factor could be osteomalacia, a condition observed in patients with beta-thalassemia (58). It may affect the degree of bone mineralization but not the trabecular microstructure. Therefore, the TBS -which reflects the degree of trabecular deterioration- appears to be not affected.

The differences observed compared with previous studies likely reflect heterogeneity in patient populations, study designs, and analytical approaches rather than true discrepancies.

In the study by Baldini et al. on TM (42), the number of patients with fractures was limited, and no significant differences were observed between those with and without fractures (1.067 ± 0.12 vs 1.034 ± 0.11, p = 0.2), with no correlation between TBS and previous fractures. The TBS may therefore be more useful as a tool for risk stratification rather than as a definitive predictor. Our findings are consistent with this interpretation but are derived from the analysis of a larger number of patients with fractures, albeit without a control group.

Teawtrakul et al. (55) analyzed a mixed population of patients with transfusion-dependent thalassemia (TDT) and non-transfusion-dependent thalassemia (NTDT). They found that patients with a low TBS had a higher risk of vertebral fractures than did patients with low BMD, and the combination of low BMD and low TBS was significantly associated with vertebral fractures. They therefore suggested performing both TBS and BMD measurements as a screening approach for vertebral fracture risk in patients with beta-thalassemia, particularly in those with iron overload and endocrinopathies. Our study differs in patient characteristics and analytical approach, as we examined a more homogeneous cohort (all receiving regular transfusion and chelation therapy) and evaluated the presence of any fragility fracture.

In a study by Osella et al. (56) involving 82 young patients with TM, the TBS demonstrated higher sensitivity, specificity, and diagnostic accuracy than BMD and Z-scores in discriminating patients with and without vertebral deformities. Moreover, combining the TBS with either BMD or Z-scores further improved diagnostic accuracy. By contrast, our study assessed the presence of any fragility fractures (spine and non-spine) rather than vertebral deformities alone, which may partly explain the different performance of the TBS in discriminating patients with and without fractures. Additional factors, such as the younger age of Osella’s cohort, potential selection bias, and differences in methods used to record skeletal abnormalities, may have also contributed to the observed differences.

Fung et al. (59) recently explored the possible use of the TBS alone or in combination with BMD in assessing bone quality and fracture risk in patients with hemoglobinopathies, including different forms of thalassemia (β-thal, E–β thal, HbH, or HbH/Constant Spring). Their findings showed that reduced bone mass was associated with overall fracture prevalence but did not differentiate fragility fractures, whereas an abnormal TBS was strongly associated with a history of fragility fracture. The differences from our results may be largely attributable to cohort characteristics (broader spectrum of thalassemia phenotypes, younger age, different ethnic background) and methodological aspects, including data collection, fracture classification, and the use of healthy controls drawn from previous research.

In summary, our data demonstrate that the TBS may be a useful complementary tool in the assessment of bone health in patients with TM, although its capacity to discriminate between patients with and without fractures is limited. Nevertheless, the study has several limitations. First, the absence of a control group limits the definition of the TBS’s role; a control group was not included because it would be unethical to perform radiological tests in young patients without clinical indications. Second, the retrospective design meant that DXA data were acquired at different times. Third, the study population size, while relatively large given the rarity of the disease and comparable with previously cited studies, remains limited. Fourth, heterogeneity in the pattern and severity of endocrinopathies could have influenced the results.

## 5 Conclusions

Our study provides a non-invasive evaluation of bone health in a relatively large cohort of patients with TM. In line with the literature, the frequency of fractures in our patients was high and may even have been underestimated. Our findings suggest that the TBS may be a valuable, non-invasive complementary tool for assessing bone quality alongside BMD, although it does not reliably discriminate between patients with and without fractures. Further prospective, well-designed studies with larger populations are needed to clarify the role of the TBS in predicting fracture risk in this group.

## Data Availability

The original contributions presented in this study are included in this article/supplementary material; further inquiries can be directed to the corresponding author.
